# THE EFFECT OF FINANCIAL STRAIN AND LANGUAGE OF INTERVIEW ON DEPRESSIVE SYMPTOMS IN MEXICAN AMERICAN OLDER ADULTS

**DOI:** 10.1093/geroni/igad104.3634

**Published:** 2023-12-21

**Authors:** Veronica Hernandez, Lucy Nasser, Soham Al Snih

**Affiliations:** University of Texas Medical Branch, Galveston, Texas, United States; University of Texas Medical Branch, Galveston, Texas, United States; University of Texas Medical Branch, Galveston, Texas, United States

## Abstract

We used data from the Hispanic Established Population for the Epidemiological Study of the Elderly to examine the relationship of financial strain and interview language to high depressive symptoms among Mexican Americans aged ≥75 years without history of depressive symptoms at baseline over a 12-year period (2004/05-2016; N=1157). Participants scored < 16 in the Center for Epidemiologic Studies Depression Scale at baseline. Measures included age, marital status, years of formal education, body mass index, social isolation, church attendance, self-reported medical conditions, cognitive and physical function, pain, falls, disability, and hand grip strength. We used a generalized estimating equation model to estimate the odds ratio (OR) and 95% confidence interval (CI) of depressive symptoms as a function of financial strain and interview language over time, adjusting for all covariates. We found that participants experiencing financial strain and interviewed in Spanish had greater odds of experiencing high depressive symptoms (OR=2.48, 95% Cl=1.37-4.48) compared to those who were not experiencing financial strain and selected be interviewed in English. No significant association with depressive symptoms was found in participants with financial strain and interviewed in English or those without financial strain and interviewed in Spanish. In conclusion, Mexican American older adults were at a high risk for high depressive symptoms over time if they were experiencing financial strain and preferred be interviewed in Spanish. Early screening for depression may prevent or delay depressive symptoms for individuals in this population at high risk for depression.

